# Accuracy-Precision Trade-off in Human Sound Localisation

**DOI:** 10.1038/s41598-018-34512-6

**Published:** 2018-11-06

**Authors:** Rachel Ege, A. John Van Opstal, Marc M. Van Wanrooij

**Affiliations:** Radboud University, Donders Institute for Brain, Cognition and Behaviour, Department of Biophysics, Heyendaalseweg 135, 6525 AJ Nijmegen, The Netherlands

## Abstract

Sensory representations are typically endowed with intrinsic noise, leading to variability and inaccuracies in perceptual responses. The Bayesian framework accounts for an optimal strategy to deal with sensory-motor uncertainty, by combining the noisy sensory input with prior information regarding the distribution of stimulus properties. The maximum-a-posteriori (MAP) estimate selects the perceptual response from the peak (mode) of the resulting posterior distribution that ensure optimal accuracy-precision trade-off when the underlying distributions are Gaussians (minimal mean-squared error, with minimum response variability). We tested this model on human eye- movement responses toward broadband sounds, masked by various levels of background noise, and for head movements to sounds with poor spectral content. We report that the response gain (accuracy) and variability (precision) of the elevation response components changed systematically with the signal-to-noise ratio of the target sound: gains were high for high SNRs and decreased for low SNRs. In contrast, the azimuth response components maintained high gains for all conditions, as predicted by maximum-likelihood estimation. However, we found that the elevation data did not follow the MAP prediction. Instead, results were better described by an alternative decision strategy, in which the response results from taking a random sample from the posterior in each trial. We discuss two potential implementations of a simple posterior sampling scheme in the auditory system that account for the results and argue that although the observed response strategies for azimuth and elevation are sub-optimal with respect to their variability, it allows the auditory system to actively explore the environment in the absence of adequate sensory evidence.

## Introduction

To estimate a sound’s direction, the auditory system has to process several implicit acoustic cues that arise from the complex, frequency-dependent interaction of sound waves with the head and pinnae: interaural differences in arrival time (so-called ITDs) and level (ILDs) specify directions in the horizontal plane (azimuth angle, α). Although these cues are highly reliable and robust, they cannot uniquely specify a sound’s direction in space, as all locations on the so-called interaural ‘cone of confusion’ lead to identical ILDs and ITDs^1^. For example, all sounds presented in the midsagittal plane of the head yield ILD = ITD = 0. Thus, to disambiguate the cone of confusion, the auditory system should identify the sound’s elevation angle too. Acoustic diffraction, reflection and interference patterns that arise within the pinna cavities are known to yield idiosyncratic, complex spectral-shape cues that uniquely encode directions in the median plane (elevation angle, ε) for broadband sounds containing frequencies exceeding about 3–4 kHz^2^. These direction-dependent acoustic pinna filters, which contain specific patterns of amplifications and attenuations that vary systematically with the elevation angle, are known as the head-related transfer functions, or HRTFs^1–10^ and are usually described by their amplitude frequency characteristics, *H(f*, *ε*).

However, it has been argued that the estimation of the elevation angle is an ill-posed problem, as the acoustic sensory spectrum at the eardrum, *S(f*, *ε*_*T*_), caused by a target sound at elevation angle *ε*_*T*_, always results from a multiplicative combination of two unknowns: the actual source spectrum, T(f), and the particular direction-dependent pinna filter^4,8–10^:

1 $$S(f;{\varepsilon }_{T})=H(f;{\varepsilon }_{T})\cdot T(f)$$

**Figure 1:**
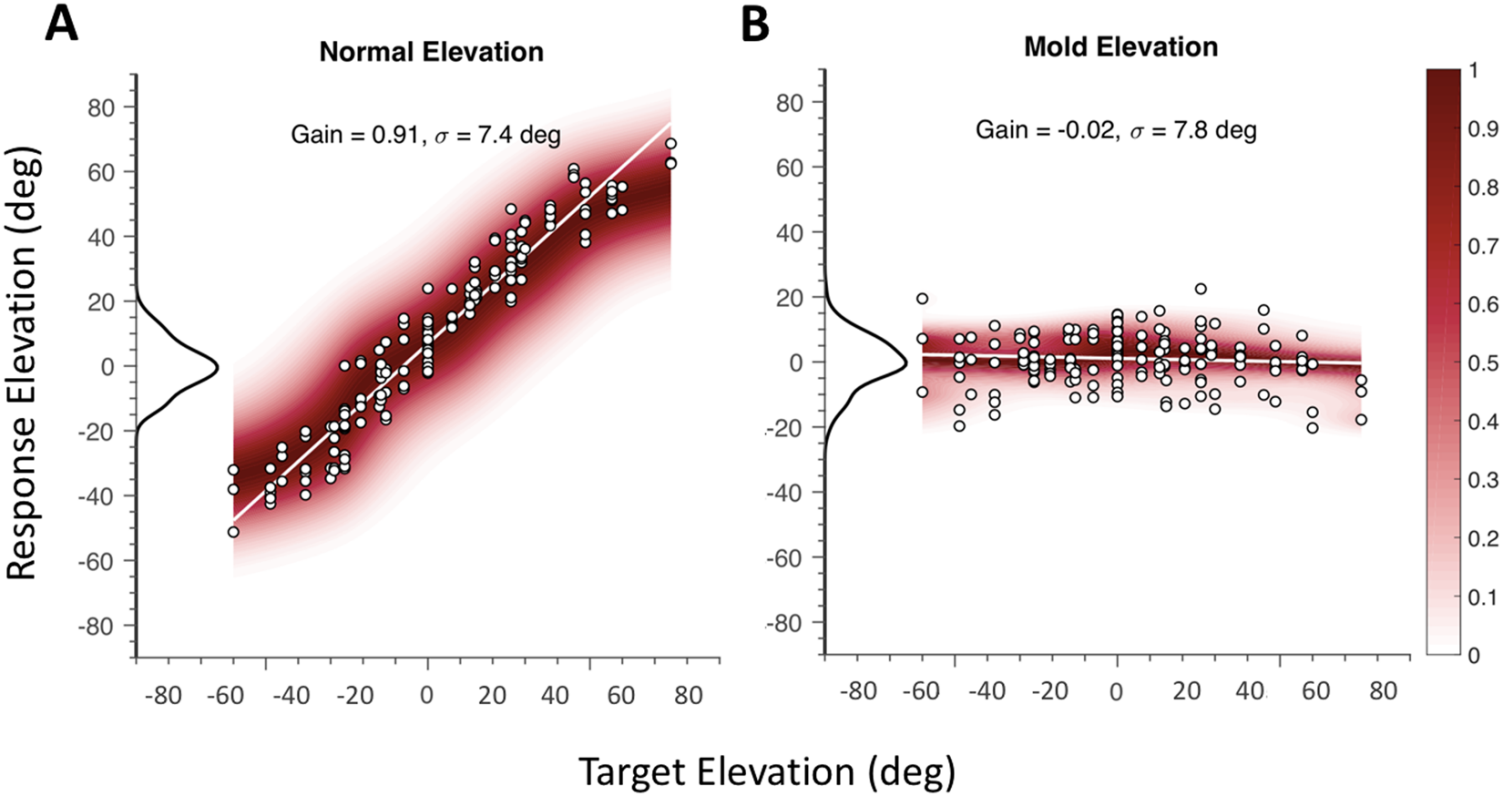
Elevation components of head-saccades to broadband sounds presented in the 2D frontal hemifield (α ∈ [−90°, +90°] and ε ∈ [−55°, +80°]). Colour code: normalized response density around the regression line. (**A**) Normal hearing. Note the high response gain and constant variability across the response range. The response distribution around the regression line is approximately Gaussian (inset left). (**B**) Localisation immediately after inserting moulds in both pinnae. Data from one subject, for whom each mould produced a comparable ipsilateral localisation deficit in elevation (but see^9^ for a more comprehensive analysis). Although these responses have zero gain, their variance is similar as for normal broad-band hearing (see Discussion). The azimuth response components for both conditions had a high gain and low variability (not shown). Red shading: data probability around the optimal fit.

As this entwined convolution provides only one equation with two unknowns, the elevation angle cannot be extracted from the sensory input with any certainty, and therefore the auditory system can never be sure about the true sound location. Yet, normal-hearing listeners localise most broadband sounds in all directions with considerable accuracy and precision^4,8,9,11,12^. Moreover, experiments under perturbed hearing conditions show invariably that the azimuth and elevation components are extracted by independent neural pathways (see, e.g.^6,8,9,12^ and Fig. 1C).

We have hypothesized that the auditory system may adopt two prior assumptions to cope with the elevation estimation problem: (i) the HRTFs are unique for each elevation angle (i.e., a pinna prior on spectral filters), and (ii) source spectra do not resemble any of the pinna filters (a spectral prior on natural sounds^8,13^. We showed that if both requirements are met, and the system would cross-correlate the sensory spectrum, as measured by the auditory nerve/cochlear nucleus, with all learned and stored spectral pinna-filter representations, the result would be a function of elevation. In the absence of noise in the sensory representations and measurements, this cross-correlation function will always peak at the veridical target elevation angle^6,8^.

The (rectified) entries of the cross-correlation vector may be interpreted as likelihoods of potential target elevations, which depend on the true stimulus location at ε_T_, represented by L(ε|ε_T_). Selecting its peak could therefore be seen as a maximum likelihood estimation (MLE) problem.

In reality, however, there will be internal noise and uncertainty in the processing chain. As a result, the cross-correlation function in a given trial could peak at a different internal elevation estimate than the true target location, e.g. at elevation ε* (see below). Yet, across many trials, the MLE will scatter around the true target location, with a variability that reflects the amount of noise in the system. This simple model predicts accurate sound-localisation performance to a wide variety of sounds for simple (single-target) listening conditions^8^.

However, for more challenging everyday listening conditions, target uncertainty may become considerable, and the MLE model could lead to large localisation errors and increased variability^8,14^. To ensure an optimal strategy for all listening conditions, the estimation process is therefore thought to involve the contribution from additional assumptions about the spatial distribution of potential targets (a spatial prior), P(α_T_,ε_T_). In that case, Bayes’ rule transforms the likelihood functions for azimuth and elevation (the sensory evidence) into more precise posterior distributions (see below), which specify the probability to localise the target at a given azimuth and elevation. For Gaussian probability distributions, the optimal localisation response, which yields minimal mean-squared localisation errors and variability across trials, is then obtained by selecting the location that maximizes the posteriors. This decision strategy is known as the maximum-a-posteriori (MAP) estimate^15,16^.

To illustrate some interesting properties of the elevation estimation process, Fig. 1 presents two examples of human sound-localisation responses, as measured in our lab under open-loop hearing conditions (i.e., short stimuli of 150 ms, presented in complete darkness, without any visual, or other modes of feedback about performance). The figure shows the elevation components of sound-evoked head-orienting saccades. Broadband sounds (bandwidth 0.2–20 kHz) were presented throughout the two-dimensional frontal hemifield for two different situations: normal hearing (Fig. 1A), and after inserting moulds into the concha of the pinnae that perturb the original spectral cues (Fig. 1B).

Several aspects of these response data are worth noting: (i) for normal hearing of BB noises (Fig. 1A), localisation in elevation is accurate (high response gain, slope ~0.9), and relatively precise (σ ≈7.5 deg). (ii) The scatter around the optimal linear fit is nearly normally distributed and does not vary appreciably with response eccentricity (suggesting additive, rather than multiplicative noise). (iii) With binaural moulds, the spectral cues of the ears are heavily perturbed, but the response variance can be similar to that of normal hearing. (iv) For both conditions, the azimuth response components were accurate (gains close to one) and precise (low variability), emphasising the independence of the azimuth and elevation pathways (not shown here, but see^4,6,8,9,11–13^.

Inspired by the localisation data for normal and perturbed hearing conditions such as in Fig. 1, and reported by numerous studies in the literature^1,3–14,17^ we here consider the hypothesis of a Bayesian MAP estimator for sound-source azimuth and elevation. We will assume that the internal prior emphasizes directions around the horizon^17^. That is, for elevation, the prior has a mean around zero and a restricted variance, whereas for azimuth the prior is assumed to be much broader, and close to uniform (i.e., all azimuth directions are nearly equally likely):

2 $$\begin{array}{c}P({\alpha }_{T},\,{\varepsilon }_{T})\propto \exp (-\frac{1}{2}{(\frac{{\alpha }_{T}}{{\sigma }_{P,\alpha }})}^{2})\cdot \exp (-\frac{1}{2}{(\frac{{\varepsilon }_{T}}{{\sigma }_{P,\varepsilon }})}^{2})\\ \,{\rm{with}}\,{\sigma }_{P,\alpha }\gg {\sigma }_{P,\varepsilon }\end{array}$$

**Figure 2:**
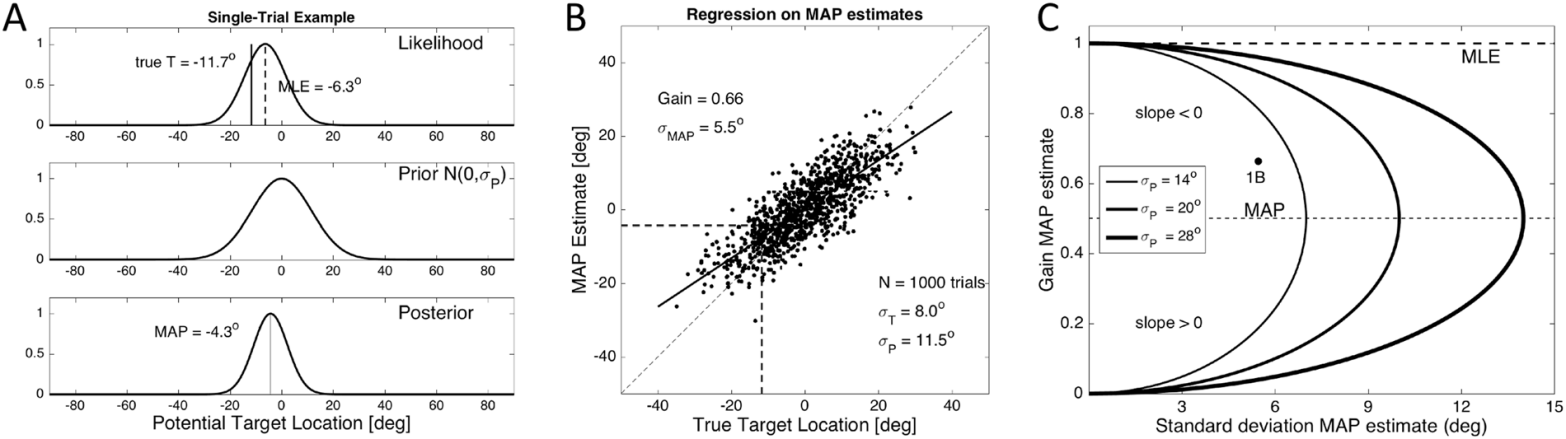
(**A**) From target presentation to MAP estimate for a single trial. The true target location (here at *ε*_*T*_ = −11.7 deg) is endowed with noise, leading to a noisy likelihood function (here with σ_T_ = 8 deg). The peak of this function is at ${\varepsilon }_{n}^{\ast }=$ −6.3 deg, which is the MLE for this trial. Combining the likelihood with the prior (zero mean, σ_P_ = 11.5 deg) leads to the posterior distribution for this trial, which has its maximum at *ε*_*MAP*_ = −43 deg. (**B**) Regression analysis on 1000 randomly selected trials with targets between −35 and +35 deg. The dashed lines point to the result of the trial in (**A**). The gain of the MAP estimates is 0.66, whereas the standard deviation of the residuals is 5.5 deg. (**C**) Predictions of the Bayesian MAP model (Eqn. 9) for the relationship between response accuracy (ordinate, gain) and precision (abscissa, standard deviation of regression residuals) for three different priors (see legend) and the simulated sensory noise ranging from σ_T_ = 0 to 100 deg. Maximal response variability is obtained for G_MAP_ = 0.5, and is given by σ_MAP,MAX_ = σ_P_/2. At this point, the slope of the curves is infinite. For G_MAP_ < 0.5 the slope is positive, whereas for G_MAP_ > 0.5 it is negative (Supplementary Material S1). MLE: maximum likelihood estimate (σ_P_ = ∞; dashed line). Filled dot ‘1B’: the regression result of panel (B).

Figure 2 illustrates the underlying statistical model for sound-localisation responses in elevation, simulated under varying noise conditions (see Supplemental Material S4). In Fig. 2A, we present the model’s mechanism for a single trial, where we took *σ*_*T*_ = 8.0 deg, and *σ*_*P*_ = 11.5 deg. In Fig. 2B the simulation was repeated for 1000 trials, with a regression analysis on the predicted responses, whereas in Fig. 2C we show how the regression results (slope and scatter around the best-fit line) are expected to vary for different noise conditions. The following presents and derives the relevant expressions underlying these simulations.

We adopted simple Gaussian models for the internal sensory noise (Eqn. 3; see Fig. 1) and priors (Eqn. 2). Suppose that the uncertainty about the true target location is described by Gaussian additive (static) noise, *η*, with zero mean and variance ${\sigma }_{T}^{2}$. Presentation of a target at *ε*_*T*_ in trial *n* then yields a Gaussian likelihood function with its mean at., where η_n_ (in deg) is a random noise sample (Fig. 2A, top):

3 $$L(\varepsilon |{\varepsilon }_{n}^{\ast }) \sim \exp (-\frac{{(\varepsilon -{\varepsilon }_{n}^{\ast })}^{2}}{2{\sigma }_{T}^{2}})$$

Here, ${\varepsilon }_{n}^{\ast }$ corresponds to the maximum likelihood estimate (MLE) of the target’s elevation, which for a given trial will typically differ from the true target direction. For example, in Fig. 2A (top) the target was presented at ε_T_ = −11.7 deg, but the MLE was obtained for ${\varepsilon }_{n}^{\ast }=-\,6.3$ deg. Across many trials, the MLE will scatter around the true target location $({\rm{i}}{\rm{.e}}.,\,\mathrm{mean}\,\overline{{\varepsilon }_{n}^{\ast }}={\varepsilon }_{T})$with variance ${\sigma }_{T}^{2}$.

The simulations assumed that the prior for potential elevations is normally distributed around the horizontal plane (i.e., mean zero), with variance ${\sigma }_{P}^{2}$ (Fig. 2A, center). It then follows that the posterior elevation for a single trial follows from Bayes’ rule as the product of the likelihood and prior distributions:

4 $$POST({\varepsilon }_{n}^{\ast }|\varepsilon ) \sim L(\varepsilon |{\varepsilon }_{n}^{\ast })\cdot P({\varepsilon }_{n}^{\ast }) \sim \exp (-\frac{{({\varepsilon }_{n}^{\ast }-{\mu }_{POST})}^{2}}{2{\sigma }_{POST}^{2}})$$

The trial’s posterior is a Gaussian, for which mean, *μ*_*POST*,*n*_, and standard deviation, σ_*POST*_, are given by^15–24^ Fig. 2A, bottom):

5 $${\mu }_{POST,n}=\,\frac{1}{1+\frac{{\sigma }_{T}^{2}}{{\sigma }_{P}^{2}}}\cdot {\varepsilon }_{n}^{\ast }\,{\rm{and}}\,{\sigma }_{POST}^{2}=\frac{{\sigma }_{T}^{2}}{(1+\frac{{\sigma }_{T}^{2}}{{\sigma }_{P}^{2}})}$$

The MAP decision rule takes the trial-by-trial estimate for the target location at the posterior’s maximum, which for the assumed Gaussian distributions equals the posterior’s mean:

6 $${\varepsilon }_{MAP,n}={\rm{argma}}{x}_{\varepsilon }[POST({\varepsilon }_{n}^{\ast }|\varepsilon )]={\mu }_{POST,n}$$

As the MLE is inherently stochastic, the posterior’s mean varies from trial to trial too. Therefore, the true target location, ${\varepsilon }_{T}$, cannot be inferred from the posterior on the basis of a single trial. In the example of Fig. 2A (bottom), the posterior scatters around *μ*_*POST*,*n*_ = −4.3 deg (the MAP estimate for this trial), with a standard deviation of *σ*_*POST*_ = 6.5 deg, which is smaller than the sensory noise and the prior width.

The result of 1000 simulated trials at randomly selected locations over a range of [−35, +35] deg is presented in Fig. 2B. It shows the 1000 MAP estimates as a function of the true target location, together with the linear regression result on the predicted responses.

When the auditory system adheres to the MAP decision rule of Eqn. 6 and the underlying distributions are all Gaussian, its responses will be normally distributed too^15^, with mean and variance given by:

7 $${\varepsilon }_{MAP}=\overline{{\mu }_{POST}}=\frac{1}{1+\frac{{\sigma }_{T}^{2}}{{\sigma }_{P}^{2}}}\cdot {\varepsilon }_{T}\,{\rm{and}}\,{\sigma }_{MAP}^{2}=\frac{{\sigma }_{T}^{2}}{{(1+\frac{{\sigma }_{T}^{2}}{{\sigma }_{P}^{2}})}^{2}}$$

This predicted response distribution has the same mean, but it is more precise than the posterior (i.e., σ_*MAP*_ < σ_*POST*_* < min(*σ_*P*_, σ_T_)). If we determine the stimulus-response relationship for this optimal Bayesian estimate, the predicted response gain (i.e., the slope of the best-fit regression line) is:

8 $${G}_{MAP}\equiv \frac{{\varepsilon }_{MAP}}{{\varepsilon }_{T}}=\frac{1}{1+\frac{{\sigma }_{T}^{2}}{{\sigma }_{P}^{2}}},\,\,\mathrm{from}\,\,\mathrm{which}\,\,{\sigma }_{MAP}^{2}={G}_{MAP}^{2}\cdot {\sigma }_{T}^{2}$$

where the right-hand side follows immediately from Eqn. 7. In the example of Fig. 2B the measured slope of the optimal regression line (G_MAP_ = 0.66) corresponds well to Eqn. 8 (G_MAP_ = 0.67) for the example values of the simulation (*σ*_*T*_ = 8, *σ*_*P*_ = 11.5). Also, the response variance around the regression line (σ_MAP_ = 5.5 deg) is accurately predicted by Eqn. 8 (σ_MAP_ = 5.4 deg).

By eliminating the (unknown) variance of the sensory noise, ${\sigma }_{T}^{2}$, we obtain the following accuracy-precision relationship between response gain and variance:

9 $${\sigma }_{MAP}^{2}={G}_{MAP}\cdot (1-{G}_{MAP})\cdot {\sigma }_{P}^{2\,}$$

In Eqn. 9, the variance of the system’s prior acts as the only free parameter of the MAP model.

Figure 2C shows the predictions of the MAP strategy (Eqn. 9) for three different priors, and for the MLE (which corresponds to the case of σ_P_ → ∞, i.e., a uniform prior). The curves show the optimal response behaviour for different sensory conditions, in which the target uncertainty, σ_T_, systematically varied from σ_T_ = 0 (top left of the curves) to σ_T_ = σ_P_ (the maximum extent of the curves, to σ_T_ = infinity (bottom left of the curves).

From Eqns 8 and 9 one finds that the predicted response variance for the MAP model will be zero for two conditions: when G_MAP_ = 1.0, which is obtained when there is no sensory uncertainty (i.e., when σ_T_ = 0 in Eqn. 7; see Fig. S3 in Supplementary material), or G_MAP_ = 0, which occurs when the sensory uncertainty approaches ∞ (i.e., there is no sensory information about the target at all; see Fig. S1). In the latter case, the posterior is entirely determined by the spatial prior, so that the MAP estimate corresponds to the prior’s mean at zero. In other words, such a decision strategy will not generate a response at all in the absence of sensory evidence. Thus, the subject would keep looking at straight ahead (which is the assumed prior’s mode).

In this paper, we tested these predictions by analysing the azimuth and elevation data obtained from two different open-loop sound-localisation experiments, carried out over different target ranges, and for different motor behaviours.

In the first experiment, broadband buzzer sounds had been presented within the two-dimensional (azimuth, elevation) oculomotor range [−35, +35] deg, at different signal-to-noise ratios (SNR) with respect to a broadband (GWN) auditory background, while a visual background of dim LEDs in the laboratory room provided explicit spatial information regarding potential target locations (Supplemental Material, Fig. S5).

From a second experiment, we quantified the head-orienting responses of listeners to low-pass filtered noises with a cut-off at either 1.5 or 3 kHz, presented over the entire frontal hemifield. As these sounds contained adequate binaural ILD and ITD cues, they can be accurately localised in azimuth. However, because human HRTFs do not vary for frequencies below 3 kHz, these sounds lack any sensory information regarding the target’s elevation direction. According to the Bayesian model, the elevation responses to such stimuli should be fully dominated by the adopted prior (illustrated in Fig. S1 for the MAP model of Eqn. 9).

## Results

### Stimulus-response relations

**Figure 3:**
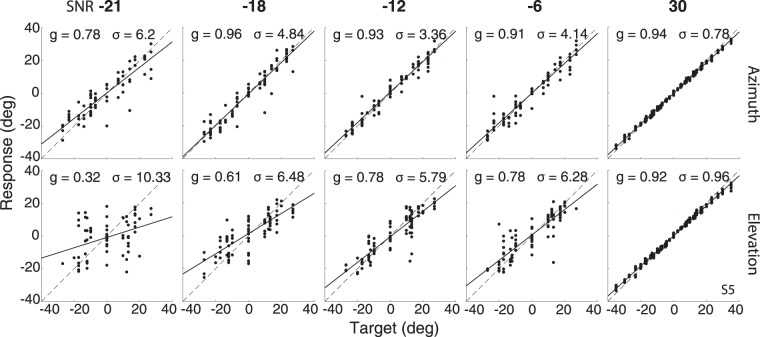
Stimulus-response data. Influence of the SNR on sound-localisation responses of subject S5 in azimuth (top row) and elevation (bottom row). Note that for elevation the variability increases strongly with decreasing SNR, and the response gain decreases. In contrast, the azimuth responses are much more robust against low SNR’s.

Figure 3 shows the azimuth (top) and elevation (bottom) stimulus-response relationships of participant S5 for five different SNRs. The fitted gains and residual standard deviations are indicated in each panel. Note that at the highest SNR (right-hand column), the responses are both accurate (high gain) and precise (low variability), for both response components. Already at a SNR of −6 dB the influence of background noise on the localisation responses becomes evident. For both components the response variability increased, and the gains lowered, although the effect is clearly more pronounced for the elevation data than for the azimuth data. These effects persisted for the lower SNRs: at the lowest SNR of −21 dB, the azimuth responses become comparable to the elevation responses at −6 dB (gain 0.78 and standard deviation 6.2 deg). The effect of a low SNR on the elevation response components is quite dramatic, as the gain dropped to a mere 0.32, with a standard deviation that exceeded 10 deg. These results therefore show that the inclusion of background noise had a strong effect on the accuracy and precision of the sound-localisation responses in elevation.

### Summary SNR and low-pass results

**Figure 4:**
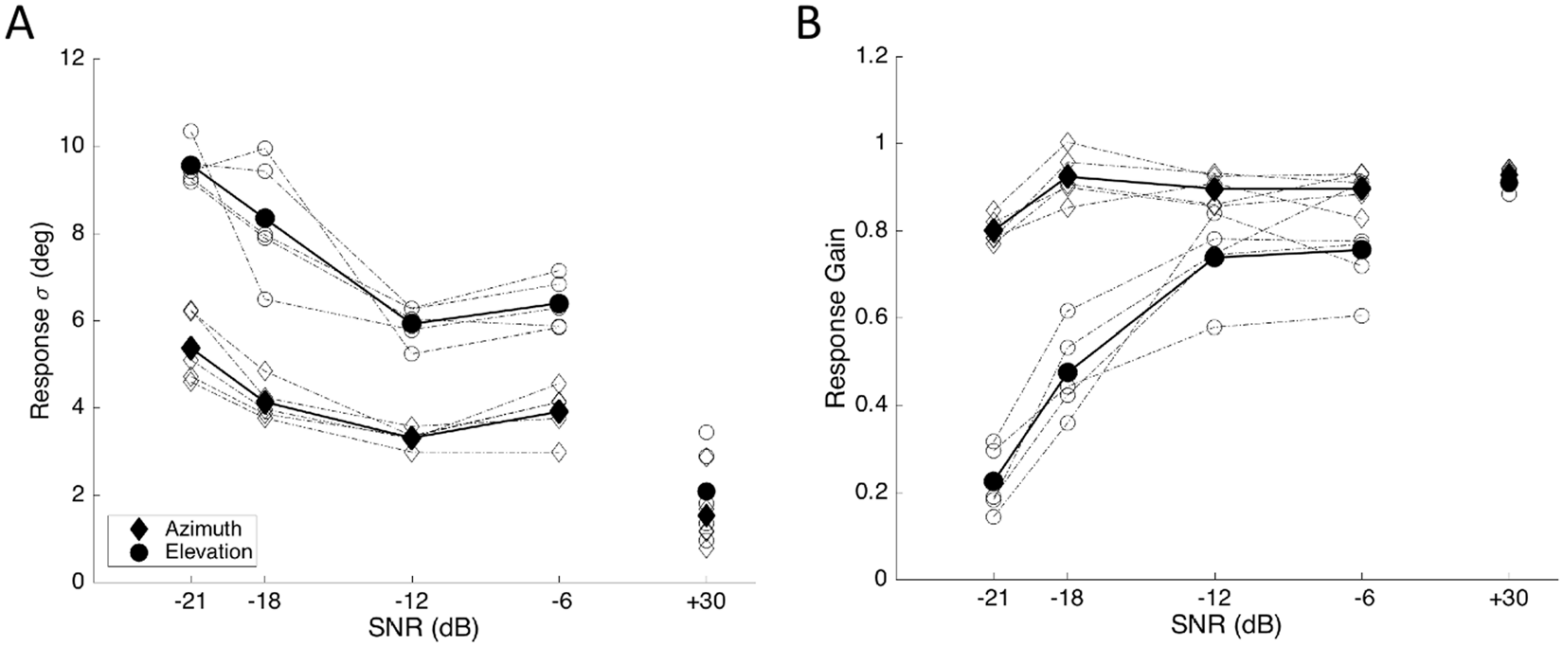
Summary of the localisation results in noise. (**A**) Standard deviation (Eqn. 15) of the response residues as a function of the SNR of the sounds, for azimuth (diamonds) and elevation (circles), for all 5 subjects (dash-dotted lines; black thick lines and filled symbols: means). (**B**) Localisation gains of the azimuth and elevation response components as a function of SNR. Note the clearly different behaviours for the azimuth (gain ~ constant) vs. elevation components for all subjects (after^27^).

Figure 4 summarizes the regression results for all participants in this experiment. Figure 4A shows that for all subjects the response variability increased with a decrease in SNR, and that this effect was stronger for the elevation response components than for the azimuth components. Figure 4B shows the systematic effects of the SNR on the response gains for the two components. As reported in other studies too, the elevation response gain is more vulnerable to background noise than the azimuth response gain^25–27^. Whereas the former already started to drop significantly at SNR = −6 dB, the latter maintained a high value up to SNR = −18 dB. These different characteristics underscore the independent neural processing pathways for the azimuth (binaural difference cues) and elevation (monaural spectral cues) target components.

Although the azimuth and elevation data seem to follow different behaviours in response to a varying SNR, the statistical model described in the Introduction (MAP) suggests that the changes in gain and response variability are coupled through Eqn. 9, regardless of the target direction (although the underlying spatial priors for the two directional components may be quite different; Eqn. 2).

**Figure 5:**
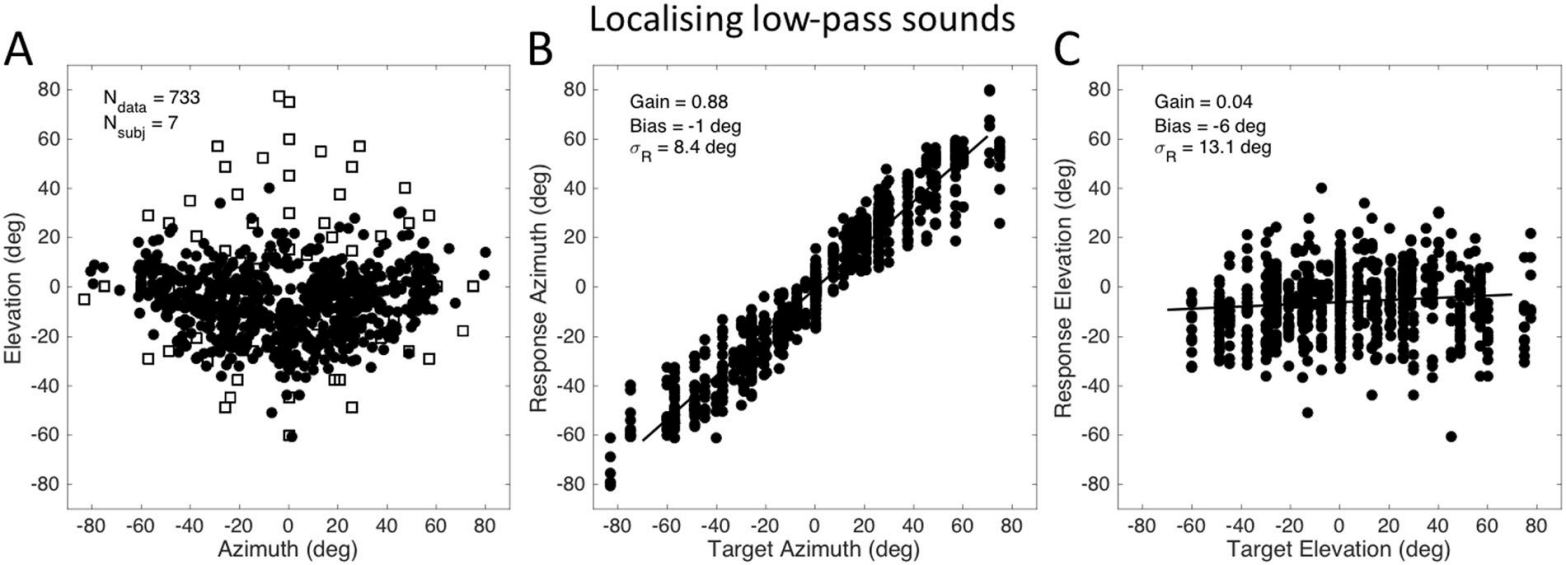
(**A**) Localisation of low-pass filtered sounds (<1.5 kHz for 4 subjects, <3 kHz for 3 subjects) over a target range of [−85, +85] deg in azimuth, and [−50, +85] deg in elevation (open squares). Filled dots: head-movement end points, pooled for seven subjects (N = 733). Subjects localised all low-pass sounds around the horizon, although sounds were interleaved with well-localisable broadband and high-pass (>3 kHz) filtered sounds. (**B**) Localisation in azimuth is accurate, with a high gain. (**C**) Localisation of elevation is impossible because low-pass sounds lack spectral cues (gain zero, bias near zero). The standard deviation of the response elevation is 13.1 deg, and results to be similar to the eye-movements in a noisy background with visual landmarks (Figs 3 and 4A).

Figure 5 shows the pooled head-movement responses (N = 733) from seven subjects to the low-pass filtered noises across the frontal hemifield. From Fig. 5A it is immediately clear that the responses are distributed around the horizontal plane, although the elevation target range was from −55 to +85 deg. The stimulus-response relation for the azimuth components (Fig. 5B) reveal a high accuracy (gain 0.9). The elevation components, however, have a response gain that is indistinguishable from zero (Fig. 5C). The response variability of the elevation responses is about 13 deg. Note that although the Bayesian model predicts that in the case of no sensory evidence the posterior equals the prior distribution, the MAP decision model would predict a very low response variability (approaching zero; Eqn. 9, Figs 2C and S1). Thus, these low-pass data do not seem to support a MAP decision strategy. The individual results from all seven subjects are provided in the Supplemental Information S7.

### Model testing

**Figure 6:**
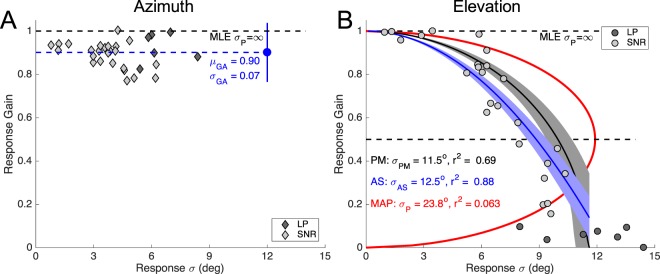
Response gain as a function of the response standard deviation. Test of the different models for (**A**) azimuth, and (**B**) elevation response components, pooled for the SNR and low-pass experiments (data from 11 subjects; N = 32 per component; one subject excluded, see Methods). (**A**) Azimuth responses invariably have a high gain, regardless of the stimulus conditions, and are best described by a constant gain around 0.9 (MLE). (**B**) The elevation responses show a fundamentally different behaviour. The data in panel B were fitted with Eqn. 9 (MAP model, red line, for which the average prediction error is zero), Eqn. 10 (AS model, blue, with standard deviation of the optimal fit in shading), and Eqn. 11 (PM model, black, shading: standard deviation). Note that all three models have the same free parameter: σ_P_. The PM and AS models both outperform the MAP decision strategy by far.

Figure 6 plots the response gains for azimuth (Fig. 6A) and elevation (Fig. 6B) against the residual standard deviations, pooled for all stimulus conditions and subjects (N = 30 points). The prediction of the MLE (at gain = 1.0) is indicated as well and shows that this model is inadequate to explain the observed behaviour of the elevation response gains but may be the best characterisation for the azimuth response components (Fig. 6A; mean gain 0.9 ± 0.07). The horizontal black dashed line in Fig. 6B at G = 0.5 intersects the data at approximately σ_res_~7–8 deg, which, according to the MAP model would correspond to a spatial prior with σ_P_~14–16 deg. The red curve shows the best-fit MAP prediction, according to Eqn. 9, for which we obtained σ_P_ = 23.8 deg. Clearly, the data do not follow the prediction of the MAP model, as for gains <0.5 the gain - variability relation in the data should have a positive slope. Instead, all data appear to follow a monotonic relation with a negative slope. As a result, the coefficient of determination between data and MAP model prediction is very low: *r*^2^ = 0.063.

The solid blue line through the data represents the best-fit parabolic relation, according to Eqn. 10 described in the Discussion (the adaptive sampling scheme, or AS). This curve intersects the G_OPT_ = 0 axis at σ_P_ = 12.5 deg. The coefficient of determination for the AS model is *r*^2^ = 0.88, which is by far better than the MAP prediction (z = 5.7; p < 10^−5^).

The black-dashed curve corresponds to Eqn. 11 in the Discussion (the posterior matching scheme, or PM), which crosses the *G*_*OPT*_ = 0 axis at *σ*_*P*_ = 11.5 deg. The coefficient of determination for the PM model is *r*^2^ = 0.69, which outperforms the MAP model too (z = 3.6; p = 0.0002). The correlation coefficient for the AS model (r = 0.94) is significantly higher (p = 0.01) than for the PM model (r = 0.83). Thus, of the four models, the AS scheme seems to predict the elevation data best.

## Discussion

### Summary

Our analysis shows that to estimate the sound’s elevation angle, the human auditory system regulates its response gain on the basis of the (perceived) reliability of current sensory information. In the SNR experiments, the target’s reliability varied from trial to trial, and subjects could anticipate neither the location of the upcoming target, nor its SNR. We revealed a novel accuracy-precision relationship for the sound-elevation responses (Fig. 6B), in which the response accuracy, quantified by the stimulus-response gain, decreased monotonically with the variance of the response residues around the optimal regression line (precision). The data also show that the Bayesian MAP model, which yields optimal accuracy-precision trade-off when the underlying distributions are Gaussian, cannot account for the data. Especially at the lower SNRs, and for low-pass sounds, the elevation response variability should decrease at the low response gains (Fig. 2C), rather than increase (Figs 3, 4 and 6).

Note that the example data from the acute mould-perturbation experiment, shown in Fig. 1B, may seem at odds with the monotonic gain-variance relationship reported in this study, as the response gain was close to zero, with a response variance (and reaction time, not shown) that compared to optimal normal-hearing broad-band sound localisation. Indeed, these data indicate that the listener was quite certain about the perceived elevation angle, although it was entirely wrong. Note, however, that as these experiments were conducted in total darkness and without any feedback, there was no way for the listener to verify whether or not the perceived location corresponded to the veridical source direction. Below, we argue that the auditory system performs a cross-correlation between the sensory spectrum and all (stored) HRTF representations. The data in Fig. 1B then suggest that the perturbed spectral input induced likelihoods that consistently peaked around the same straight-ahead elevation for all sources (see^6,28^ for a comprehensive analysis of this idea). Thus, the statistical inference is applied to *represented* source locations, described by the cross-correlation function, or stimulus likelihood, rather than to the *actual*, physical source locations.

One may wonder whether other assumptions for the prior distribution than Gaussian could explain the data with a MAP decision rule. For example, since subjects were exposed in the SNR experiments to a dimly lit visual display that revealed the potential target range, an alternative prior could have been a uniform box distribution within the oculomotor range, i.e. P(ε*) = 1/70 for −35< ε* < +35 deg, and 0 for |ε*| > 35 deg.

In the Supplemental Material (Fig. S6) we show that such a uniform box-prior cannot account for the data either. Although the box-prior also yields monotonic relationships between the response gain and response variance, the predictions differ profoundly from the experimental data in Fig. 6B.

Note that the low-pass stimuli (Fig. 5) provide no spectral cues for elevation, although the binaural difference cues that specify source azimuth are fully present. As a result, the elevation responses would depend entirely on the adopted prior. If so, the data would suggest an elevation prior that is approximately Gaussian distributed around the horizon, with a standard deviation of about 10−12 deg (Figs 5C and 6B).

The azimuth response components followed a different strategy, in the sense that they persistently relied more on sensory evidence than the elevation system. This underscores the fact that the binaural localization cues are highly reliable for the entire acoustic frequency range and are much less vulnerable to noise perturbation than the high-frequency spectral-shape cues^25,26^. As a result, a spatial prior is expected to be much less influential for the azimuth direction. The SNR and low-pass data both support evidence for a near-uniform azimuth prior, as the response gain did not depend systematically on azimuth-response variance (Fig. 6A). Instead, the data scattered around a mean constant gain of about 0.9, which would be in line with MLE.

### Posterior sampling schemes

According to the Bayesian framework, the brain aims for a response strategy that optimizes an accuracy and precision trade-off^15,16,18–22^. The MAP decision strategy predicts that as target uncertainty increases, the gain will gradually drop to zero. In the limit of very low SNRs, for which the brain no longer obtains an adequate sensory estimate, the MAP model predicts that response gain and variability both drop to zero (Figs 2C and S1; Eqn. 9). In that case, the posterior is entirely determined by the system’s prior, and since the MAP bases its decision on the maximum of the posterior, the selected response will always be the same. In case the Gaussian prior is centred around the horizon^17^, the system’s response will always point at zero elevation. Clearly, this is not in line with experimental data on saccades, which tend to explore the environment in all directions whenever the system expects a sensory event, even when the event itself is undetectable^29,30^.

Instead of the MAP estimate, we here consider two alternative sampling strategies on the posterior distribution to account for the observations. In the first strategy, the variance of the selected responses equals the variance of the posterior, by adopting a particular sampling scheme, to be described below. This decision strategy we here notify by ‘adaptive sampling’, or AS model, for which σ_AS_ ≡ σ_POST_. In this scheme, the system takes a randomly selected sample from a restricted range around the peak of the posterior to decide on its response. Eliminating the sensory noise, σ_T_, from Eqn. 5, then predicts a universal, linear gain–variance relation, with a constant, negative, slope that is given by $-1/{\sigma }_{P}^{2}$ (Supplementary Material):

10 $${G}_{AS}=1-\frac{{\sigma }_{AS}^{2}}{{\sigma }_{P}^{2}}$$

Here, the prior’s standard deviation, σ_P_ is the only free parameter, and is obtained from the intercept of Eqn. 10 with the G_AS_ = 0 axis (Fig. 6B). It is not trivial how to estimate the posterior’s variance by means of a (random) sampling strategy, as in every trial the system produces a different posterior (see Fig. 2B). The question then is how the system could acquire this information from the trial-by-trial instantiation of the posterior distribution. A simple heuristic solution is described below.

The second sampling strategy is a uniform posterior matching (PM) scheme^31,32^ in which each trial generates a random sample taken from the entire posterior distribution (Eqn. 5) to specify the response for that trial.

To compare the emerging gain-variance relationships for the three different sampling strategies considered in this paper, we simulated the models by determining the resulting regression parameters of each strategy for a large range of noise conditions (like Fig. 2B shows for the MAP model, with σ_T_ = 8.0 deg). The Matlab code for these simulations is given in Supplemental Material S4). In all simulations, the standard deviation of the prior was fixed at σ_P_ = 11.5 deg.

**Figure 7:**
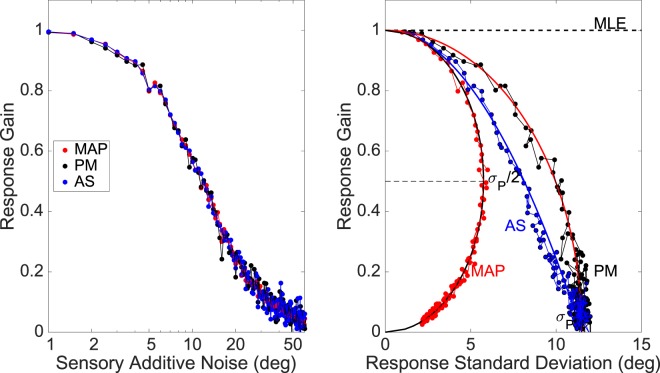
Model simulations. Random sampling of responses from the posterior distribution (PM; black) vs. MAP (red) and adaptive sampling AS (blue). (**A**) Response gain decreases with increasing additive noise (on logarithmic scale) for all sampling schemes (cf. with elevation data in Fig. 3B). (**B**) Response gain as a function of response standard deviation (on linear scale). The PM data (black) intersect the G = 0 axis at exactly the prior at σ_P_ = 11.5 deg. Red line through the PM data: Eqn. 11. The MAP variability reaches its maximum at σ_P_/2 = 5.75 deg for G = 0.5 (hor. dashed line, and black curve). Blue curve: parabolic relation (AS model, Eqn. 10), as sampled by Eqn. 12.

Figure 7 shows the simulation results for the PM model (black symbols), the AS model (blue), and the MAP estimates (red) for the different noise conditions. For comparison, the MLE is also indicated (gain = 1.0, irrespective of the sensory noise). Targets were uniformly distributed between −90 and +90 deg, and each dot in the plot corresponds to a single regression result on the basis of 1000 trials (like in Fig. 2B for MAP). The additive sensory noise on each target position had a standard deviation, σ_T_, which was varied between 1 and 60 deg, in 0.5 deg steps (119 noise conditions), i.e., ${T}_{n}^{\ast }={T}_{n}+{\eta }_{n}\,{\rm{with}}\,\eta \in N(0,\,\,{\sigma }_{T})\,{\rm{and}}\,n=1-1000.$

Figure 7A shows how the gain drops as a function of the noise: the three sampling schemes predict exactly the same behaviour, because their gains are taken from the same posterior’s mean. Figure 7B shows that the gain-variance relations, however, differ markedly for the three sampling schemes. For small amounts of noise (like observed in the azimuth responses of the SNR experiment) all three sampling schemes(and the MLE) predict very similar behaviours, which will be hard to be distinguished experimentally. However, as the sensory noise values approach the prior’s standard deviation, the curves start to deviate significantly. The blue curve in Fig. 7B shows the parabola of Eqn. 10 (AS model; cf. Fig. 6B).

Although the PM data in Fig. 5B intersect the G_OPT_ = 0 axis exactly at the standard deviation of the prior, like the AS model of Eqn. 10, it systematically overestimates the gain-specific response variance of the AS predictions at intermediate noise conditions. The PM data are well described by the following heuristic relation:

11 $${G}_{PM}=\sqrt{1-\frac{{\sigma }_{PM}^{2}}{{\sigma }_{P}^{2}}},$$

which is shown as the red curve through the PM data in Fig. 7B.

Thus, taking a random sample from the entire posterior distribution under all noise conditions produces more response variance than is actually observed in the data. Although the experimental data have a higher response variability than the MAP prediction at the same prior, they appear to fall between the PM and MAP models (Fig. 6B). The PM model ensures an optimal response gain, albeit with a higher response variability, indicative of ‘sub-optimal’ behaviour. The data suggest that the auditory system may actually outperform the PM scheme (and as such would be ‘near-optimal’), by adopting the AS sampling strategy.

The experimental data (and Eqn. 10) follow a response strategy that seems to betray a gradual transition from the optimal MAP decision at high sensory confidence levels (i.e., at low sensory noise) to full posterior random sampling at very low sensory confidence (i.e., at high noise levels). The blue symbols in Fig. 7B implement a heuristic sampling scheme, in which an estimate of the sensory noise, $\hat{\sigma }$, determined the range over which the posterior distribution was sampled around its peak to decide on the response, *R*_*AS*_:

12 $${R}_{AS}\in [{\mu }_{POST}\pm {w}_{0}\cdot \hat{\sigma }]\,{\rm{with}}\,\,{w}_{{\rm{0}}}\approx 0.9$$

At high sensory noise levels, or in the absence of sensory evidence, like observed in the low-pass data, the system samples (nearly) the entire posterior distribution, which in that case is fully dominated by the prior (Eqn. 9). For low sensory noise values, however, the response is mainly determined by the peak of the posterior (like in MAP), which is also close to the MLE prediction (Fig. S3). For intermediate sensory noise levels, the sampling width gradually increases, yielding responses that fall between these two extremes, and closely follow the prediction of Eqn. 10.

The data suggest that the auditory system may weigh its uncertainty about the sensory evidence to program its localisation response from trial to trial. The statistical model, described by Eqns 6, 10 and 12, accounts for the full behaviour of eye- and head-movement responses across a wide range of target directions, acoustic stimuli, and SNRs.

### Neural mechanisms

**Figure 8:**
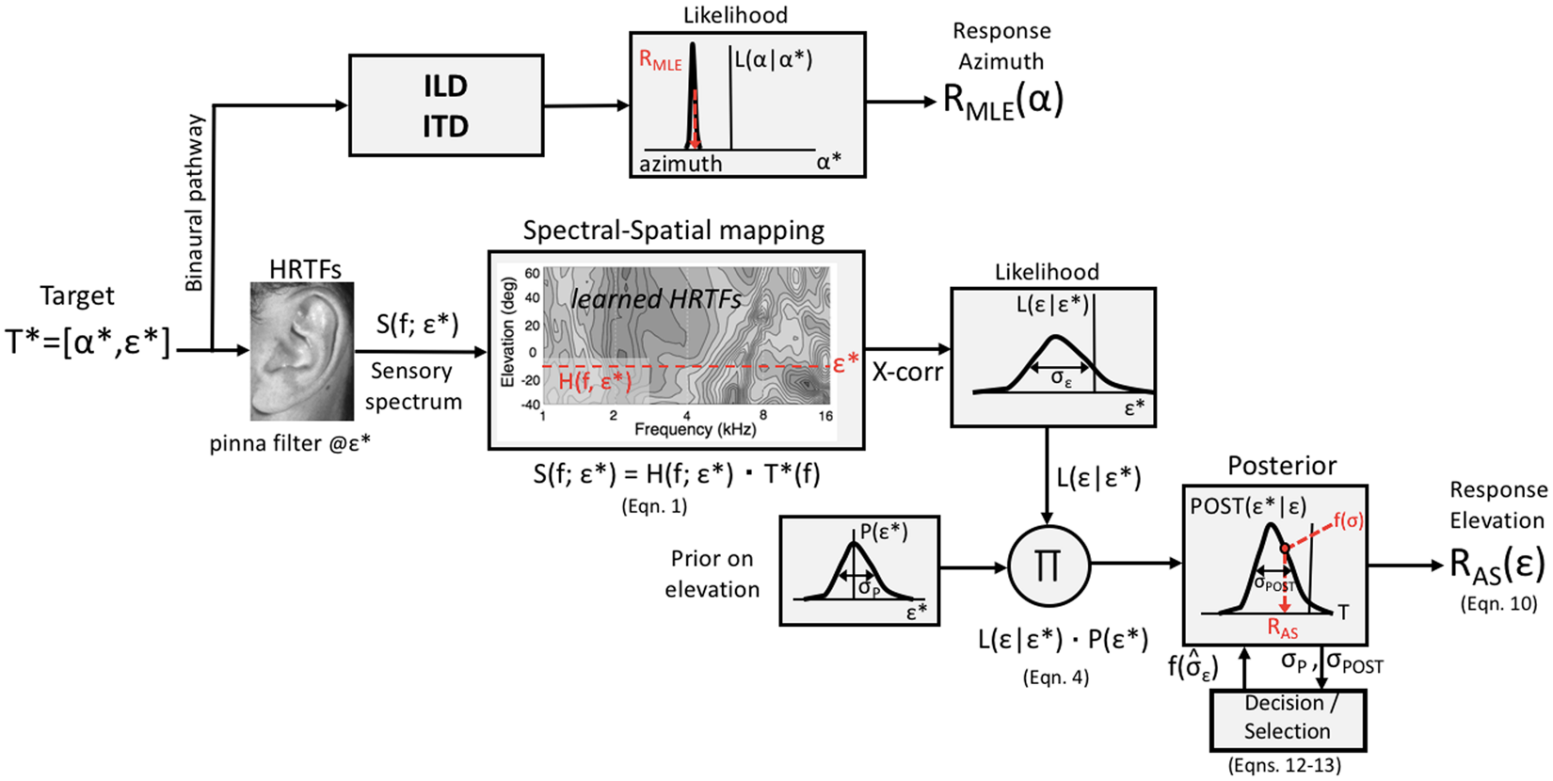
Neuro-computational model of human sound-localisation in azimuth and elevation. The coordinates are extracted by essentially independent neural pathways, as the underlying mechanisms for azimuth and elevation are profoundly different in processing and associated uncertainties. Azimuth is typically more precise than elevation (e.g., Fig. 3), yielding much narrower likelihood functions, and a better MLE. The decision/selection mechanism for elevation determines the sampling strategy on the posterior on the basis of current sensory uncertainty in the median plane (here: AS model; Eqns 12 and 13).

How could the auditory system access the relevant components and parameters of the AS model? This question concerns an internal estimate for the amount of sensory noise, σ_T_, and the posterior distribution. Figure 8 presents a computational model, adapted and extended after^8^, that explains how the auditory system could estimate the veridical direction of a sound-source in elevation and azimuth, despite the ill-posed nature of the problem (see also the Introduction, where we described the initial stages of this model), and despite internal sensory noise.

The width, σ_T_, of the likelihood function, L(ε|ε*), is assumed to provide a measure for spatial target uncertainty. Multiplication (Π) of the likelihood with the internal prior results in the posterior. In the AS sampling scheme, the sampling width is determined by a sensory-noise dependent decision stage, $f(\hat{\sigma })$, which could use the widths of the calculated posterior, σ_POST_, and internal prior, σ_P_, from:

13 $$f(\hat{\sigma }) \sim {w}_{0}\cdot \sqrt{\frac{{\sigma }_{POST}^{2}}{1-\frac{{\sigma }_{POST}^{2}}{{\sigma }_{P}^{2}}}}$$

Thus, the more the variance of the posterior approaches the prior’s variance, the larger the estimate of the sensory uncertainty, and the wider the posterior sampling range, as prescribed by Eqn. 12.

We speculate that a potential neural correlate for the posterior distribution (as the neural representation of the system’s desired motor output) could be embedded in the population activity of the motor map of the midbrain Superior Colliculus (SC). The SC population activity can be well described by a rotation-symmetric Gaussian in its motor map^33^ and could potentially represent a statistical distribution of potential responses^23^. It has been shown that the cells in the SC population together specify the response coordinates (amplitude and direction of the gaze-saccade), as well as its kinematics^34,35^. Noise can enter this population in different ways: (i) by random changes of centre and shape of the population, (ii) by variability of neural firing rates across the population, and (iii) by variability in the number of spikes of the recruited cells. Each of these factors could have a different effect on the resulting motor responses: e.g., variability in response endpoints because of (i) and (iii)^36^ and variability in movement speed because of (ii)^35^.

We have proposed that the fastest and most precise gaze shifts occur when all cells in the population synchronise their bursts^35^ and that the SC motor map as such embeds optimal speed-accuracy trade-off. This sensory-motor mechanism ensures that saccades are generated as fast and as accurately as possible, despite target uncertainty in the retinal periphery, by following the so-called nonlinear main-sequence kinematics (a saturating amplitude - peak velocity relation^35,37,38^ and a tendency to undershoot visual targets by about 10%^39^. Jitter in the timing and properties of SC bursts would thus cause gaze trajectories to deviate from a straight line, increase the saccade endpoint variability, and endow them with slower, non-optimal kinematics.

We here conjecture that the SC population could also implement (near-)optimal accuracy-precision trade-off, as forwarded in this study. The more confident the system is about the target coordinates, the more vigorous the resulting SC bursts, whereas increased uncertainty about the target would reduce vigour and synchrony among the cells. The subsequent collicular readout (brainstem/cerebellum) could derive a measure for the posterior’s variance, σ_POST_, from the intrinsic variability within the SC population^23^ and relate this to the uncertainty in the target representation to select its localization estimate.

### In conclusion

Taken together, our analysis reveals how the unique independence of the horizontal and vertical sound-localisation mechanisms, are not only processed by independent neural pathways, but may also be embedded as different strategies to deal with spatial uncertainty in the acoustic environment. Whereas the azimuth coordinate may be extracted by maximum likelihood estimation, the elevation direction appears to rely strongly on the involvement of a spatial prior. By testing eye- and head movements to different types of sounds, hearing conditions, different stimulus ranges, and with or without spatial environmental cues, the elevation data were best explained by a Gaussian spatial prior around straight ahead with a limited width of about 12 deg. The system appears to rely on a random sampling strategy from the posterior distribution, rather than on a point estimate like the MAP rule. In this way, the audio-motor system can explore the sensory environment with randomly directed orienting movements, even in the absence of a detectable sensory input signal.

## Methods

The auditory localisation data used in this study had been collected in the context of an earlier audio-visual integration study (SNR^27^; and a sound-localisation adaptation study (low-pass; in preparation). Here, we summarize the details of the experiments, as far as they are relevant for the used auditory data.

### Participants

Five adult male subjects (S1–S5) participated in the SNR experiments. All subjects had normal hearing, determined by a standard audiogram of both ears. All were experienced with eye-movement recording studies; S3 and S4 were authors of this paper, the other three participants had never been involved in sound-localisation paradigms. Seven other adult subjects (S6–S12; five male) participated in the head-orienting low-pass localisation experiments.

Prior to the experiments participants gave their written informed consent. The experimental protocols were approved by the local ethics committee of the Radboud University, Faculty of Social Sciences, nr. ECSW2016-2208-41. All experiments were conducted in accordance with the guidelines and regulations of the Radboud University.

### Localisation Paradigms

The SNR experiment consisted of the presentation of visual-only, auditory, and audio-visual trials, which were randomly interleaved. Here we report exclusively on the auditory-only trials, for which we systematically manipulated the SNR.

Each SNR trial began with the appearance of an audio-visual background (85 dimly lit green LEDs distributed across the entire stimulus range of ±35 deg in all directions, and a diffuse Gaussian white-noise acoustic background at 60 dB A-weighted (See Fig. S5 in Supplemental Material). A trial started by fixation of a central red LED at straight ahead. In the auditory trials, whenever this LED turned green, a peripheral auditory broad-band buzzer was presented for 2250 ms. The signal-to-noise ratio (SNR) for the auditory target was varied, by employing four buzzer intensities, each at equal probability (SNR = −6, −12, −18 and −21 dB) with respect to the background. In the no-background control condition, subjects localised a 60 dBA buzzer (the SNR was then +30 dB). Participants were required to localise the auditory target as fast and as accurately as possible, by making a head-fixed eye saccade to the perceived target sound. The target location was selected pseudo-randomly from one of 24 possible locations (12 directions, eccentricity = 14, 20, 27 deg) with equal probability (vs. 72 locations for the no-background control condition). Eye movements were recorded and calibrated with the scleral search-coil technique^27,40^.

Low-pass filtered sounds only contained frequencies between 0.5–3.0 kHz (S10–S12), and between 0.5–1.5 kHz (S6–S9), and were presented at an intensity of either 50, 60, or 70 dB SPL (A-weighted). The sounds were presented from randomly selected locations within the entire frontal hemifield, from one of 125 broad-range speakers that were mounted on a wire frame that spanned a globe with a radius of 1.25 m around the centre of the subject’s head. Azimuth angles in [−90, +90] deg, and elevation angles in [−55, +85] deg (see Fig. 5A and Supplemental Information S7; note that in the double-pole azimuth-elevation coordinate system, the sum of the absolute coordinate values can never exceed 90 deg). The listener responded with a rapid head saccade to the perceived target location, by pointing a head-fixed visual pointer at 40 cm in front of the nose (a red, dim laser spot projected onto a 1 cm^2^ black plate) in the perceived sound direction. This method prevented the subject from making combined eye-head movements, by keeping the eyes fixed in the head. Head orientation in space was measured with a search coil on the head within three perpendicular oscillating magnetic fields^7,12,40^.

### Regression

To determine the response accuracy and variability, we performed linear regressions on the azimuth and elevation stimulus-response components for each subject:

14 $${R}_{pred}=g\cdot T+b$$

with *R*_*pred*_ the predicted saccadic localisation response component (in deg), and *T* the actual stimulus location component (in deg, for azimuth, or elevation). Regression parameter *g* is the localisation gain (or slope, dimensionless), and *b* is the localisation bias (or offset, in deg) of the optimal fit through the component data^41^. Localisation biases were typically small (close to 0) and were as such not regarded in further analyses. Optimal regression parameters were found by minimizing the mean-squared error. When the regression gain deviated by more than 3 standard deviations from the mean, we excluded the regression result from the group analysis. This occurred for the low-pass azimuth responses of subject S8 (gain 0.59; Supplemental Information Figs S7–3).

The response variability was defined as the standard deviation, *σ*_*res*_, of the fit residuals (the mean squared errors):

15 $${\sigma }_{res}^{2}={\langle ({R}_{meas}-{R}_{pred})}^{2}\rangle $$

where *R*_*meas*_ is the measured response per trial and <*x*> is the average of *x* across trials.

## Electronic supplementary material

Supplemental Information

## Data Availability

The data sets analysed for the current study are available from the corresponding author on reasonable request.
