# Supplementary material for: Accuracy-Precision Trade-off in Human Sound Localisation
Source: Sci Rep. 2018 Nov 6;8:16399. doi: 10.1038/s41598-018-34512-6 (PMC6219530; doi:10.1038/s41598-018-34512-6)
Supplement: Supplementary file 1 — Supplemental Information [file 41598_2018_34512_MOESM1_ESM.pdf]

Accuracy-Precision Trade-off in Human Sound Localisation

Rachel Ege, A. John Van Opstal, and Marc M. Van Wanrooij

Radboud University, Donders Institute for Brain, Cognition and Behaviour,

Department of Biophysics, Heyendaalseweg 135, 6525 AJ Nijmegen, The Netherlands

Supplementary material

S1. MAP predictions for three different sensory noise conditions: $\sigma_T \sim \infty$, $\sigma_T = \sigma_{PRIOR}$, and $\sigma_T \sim 0$.

According to the MAP model, when the uncertainty in the sensory information, σ_T, is very large, the posterior distribution will become equal to the prior. As a result, the MAP prediction will point to the straight-ahead location at every trial, leading to a response gain and standard deviation of zero. Figure S1 shows a simulation of this case (a flat likelihood) in the same format as in Figure 2A,B.

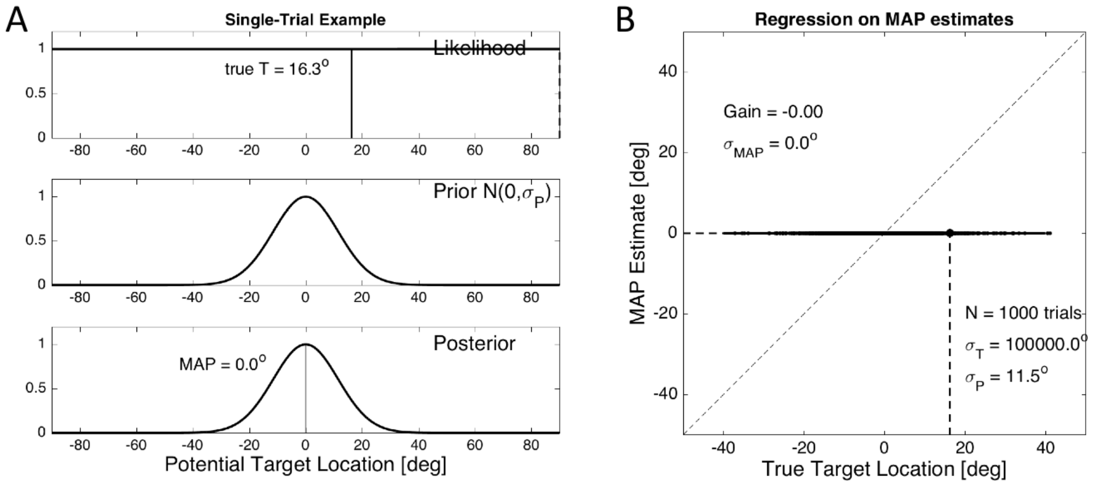

Figure S1. *(A) Simulation of the MAP estimate for a single trial. The uncertainty in the sensory estimate is 10^5 deg (infinite), yielding a flat likelihood function. The posterior distribution is identical to the prior. Thus, although the true target is presented at 16.3 deg, the MAP estimate is at 0 deg. (B) Simulation of 1000 randomly selected trials with subsequent linear regression on the MAP estimates. The result is a gain and a standard deviation of zero, as all MAP estimates are exactly at 0 deg.*

When the uncertainties in prior and likelihood are the same, the MAP model predicts averaging of the target estimates with respect to the prior mean (here, at straight ahead; response gain G_{MAP} = 0.5; Figure S2).

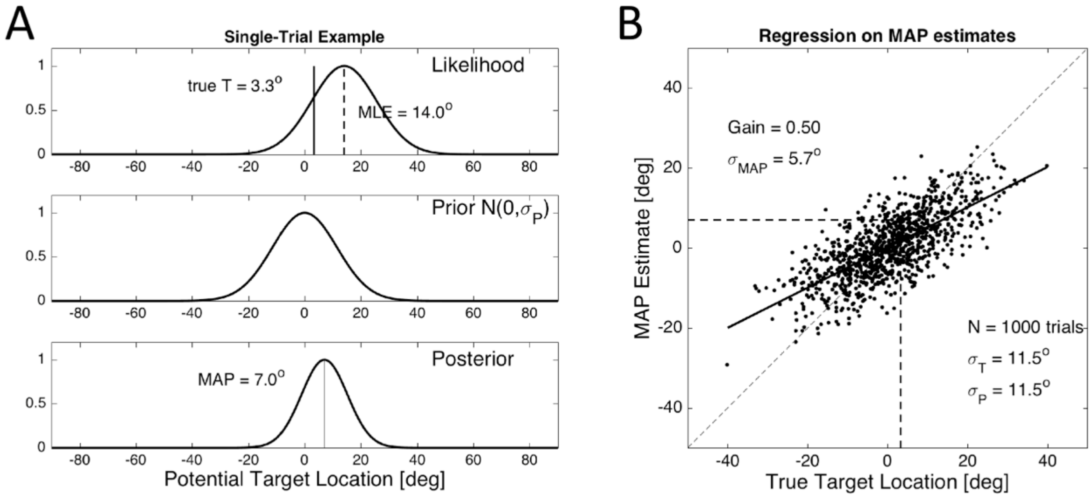

Figure S2. *(A) The uncertainty in the sensory estimate is chosen identical to that of the prior (σ_T=σ_P=11.5 deg). The posterior distribution has a standard deviation that is exactly half of the likelihood and the prior (5.75 deg). The MAP estimate is at 7 deg, which is halfway between the prior mean (0 deg) and the MLE (at 14 deg). (B) Simulation of 1000 randomly selected trials with linear regression on the MAP estimates, resulting in a response gain of 0.5, with a standard deviation of 5.7 deg = $\sigma_P/2$.*

Finally, when the sensory uncertainty is very small (here σ_T = 0.1 deg) the likelihood will fully dominate the estimate. Thus, the MAP estimates will be veridical (responses: G_{MAP}=1.0 and σ_{MAP} = 0; Figure S3).

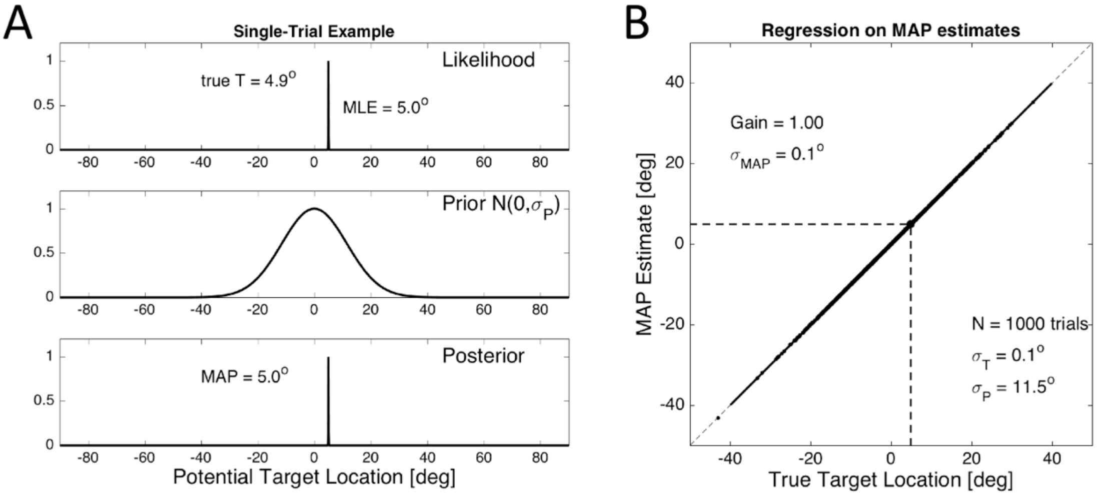

Figure S3. *(A) Uncertainty in the sensory estimate is now close to zero (0.1 deg). MLE and true target location are therefore nearly identical (5 deg). The posterior distribution has standard deviation nearly zero, and the MAP estimate now points at the true target. (B) Simulation of 1000 randomly selected trials with a subsequent linear regression on the MAP estimates. The result is a gain of 1.0, and a standard deviation of 0.1 deg.*

In Figure 2C, these three examples are found at (0, 0), (5.75, 0.5), and (0, 1), respectively. All MAP simulations were carried out with the Matlab routine of **S4**.

S2. The MAP derivative.

For the MAP model, Eqn. 6, the optimal response gain is related to the response variance by

$$G_{OPT} = \frac{1}{2} \pm \frac{1}{2}\sqrt{1-4\frac{\sigma_{MAP}^2}{\sigma_P^2}} \qquad (S1)$$

where $0 \le \sigma_{MAP} \le \sigma_P/2$ (see the semi-elliptic curves in Figs. 2, 4, and 5). For optimal gains smaller than 0.5, the slope of the curve's lower half is then given by

$$\frac{\partial G_{OPT}}{\partial \sigma_{MAP}} = \frac{2\sigma_{MAP}}{\sigma_P^2 \cdot \sqrt{1-4\frac{\sigma_{MAP}^2}{\sigma_P^2}}} > 0 \qquad (S2)$$

The slope is zero at $\sigma_{MAP}=0$ (when $G_{OPT}=0$); it approaches $+\infty$ at $\sigma_{MAP} = \sigma_P/2$, where $G_{OPT} = 0.5$.

S3. Derivation of Eqn. 10

According to the AS model, the mean and variance of the posterior distribution (Eqn. 5) directly relate to the response gain and the response variance is the same as the variance of the posterior: $\sigma_{AS}^2 = \sigma_{POST}^2$. We write the response gain (Eqn. 8) as:

$$G_{AS} = \frac{\sigma_P^2}{\sigma_T^2 + \sigma_P^2} \quad \text{from which} \quad \sigma_T^2 + \sigma_P^2 = \frac{\sigma_P^2}{G_{AS}} \qquad \text{(S3)}$$

Thus, for the variance of the posterior:

$$\sigma_{AS}^2 = \frac{\sigma_T^2\sigma_P^2}{\frac{\sigma_P^2}{G_{AS}}} = \sigma_T^2 G_{AS} \quad \text{and with } \sigma_T^2 = \frac{\sigma_P^2}{G_{AS}} - \sigma_P^2 \quad \text{follows Eqn. 10} \qquad \text{(S4)}$$

S4. Matlab code to simulate the MAP, PM and AS models (Figs. 2 and 7)

```
function [Gmap,Smap,Gpm,Spm,Gas,Sas] = posterior_sampling(sdE)

%  sdE enters the function as the sensory noise in deg (standard deviation sdE in [1 : 0.5 : 60] )
Ntr      = 1000;                 % number of trials
sdT      = 11.5;                 % Target distribution stdev, in this case it's also the stdev of the prior distribution
T        = sdT*randn(Ntr,1);     % randomly drawn targets between about -35 and +35 deg
x        =  T+sdE*randn(Ntr,1);  % 1000 noisy measurements of the targets with stdev sdE
s        = -90:0.1:90;           % stimulus axis s, of potential locations; 1801 values for the distributions, in 0.1 deg steps

%  prepare the Gaussian distributions: prior, likelihood and posterior
prior    = normpdf(s,0,sdT);     % Gaussian prior on stimulus location s, mean zero, stdev 11.5 deg
[S,X]    = meshgrid(s,x);        % S, X:  1000 x 1801 matrices: each trial gives a full distribution over 1801 points (e.g. Fig. 2A)
L        = normpdf(S,X,sdE);     % 1000 Gaussian likelihood functions (for noise sdE) given all trials on x
prior    = repmat(prior,size(L,1),1);  % repeat the prior for every trial (1000 x 1801)
post     = prior.*L;             %  posterior distributions for each individual trial (1000 x 1801)

%   MAP simulation
[~,J]    = max(post,[],2);       % the locations of posterior maxima (1000 values, one for each trial; see e.g., Fig. 2B, S1-3)
MAP      = s(J);                 % the 1000 response estimates for which the posteriors reached their maximum
b        = regstats(MAP, T, 'linear',{'beta','r'});   % linear regression performed on the 1000 trials (see e.g., Fig. 2B)
Gmap     = b.beta(2);    Smap = std(b.r);    % MAP gain and stdev of the residuals for the given target noise, sdE (data for Fig. 7)
```

```
% Posterior matching simulation: the PM model, a random sample of the posterior distribution in each trial
PM  = NaN(Ntr);
for n = 1:Ntr
     PM(n) = randpdf(post(n,:),s,[1,1]);      % draw a random sample from each of the 1000 posteriors
  end
b          = regstats(PM ,T, 'linear',{'beta','r'});
Gpm        = b.beta(2);      Spm = std(b.r);     % PM gain and stdev of the residuals (data points for Fig. 7)

% Adaptive sampling of the posterior with sampling widths depending on sdE (Eq. 11): AS model
AS = NaN(Ntr);                                   % 1000 estimates
for n = 1:Ntr
    k  = floor(J(n)-9*sdE);                      % target resolution is 0.1 deg:  is – 0.9*sdE in deg from the maximum
    m =   ceil(J(n)+9*sdE);
    smpl = s(k:m);                               % sampling width (m-k) in sensory space on the posterior depends on sdE
    post2 = post(n,k:m);                         % the partial posterior around the maximum (note: if k=m it's MAP)
    AS(n) = randpdf(post2, smpl, [1,1]);         % draw a random sample from the  partial posterior (1000 times)
 end
b          = regstats(AS, T, 'linear',{'beta','r'});
Gas        = b.beta(2);    Sas  = std(b.r);      % AS gain and stdev of the residuals (data points for Fig. 7)
```

S5. Target display of the SNR experiments

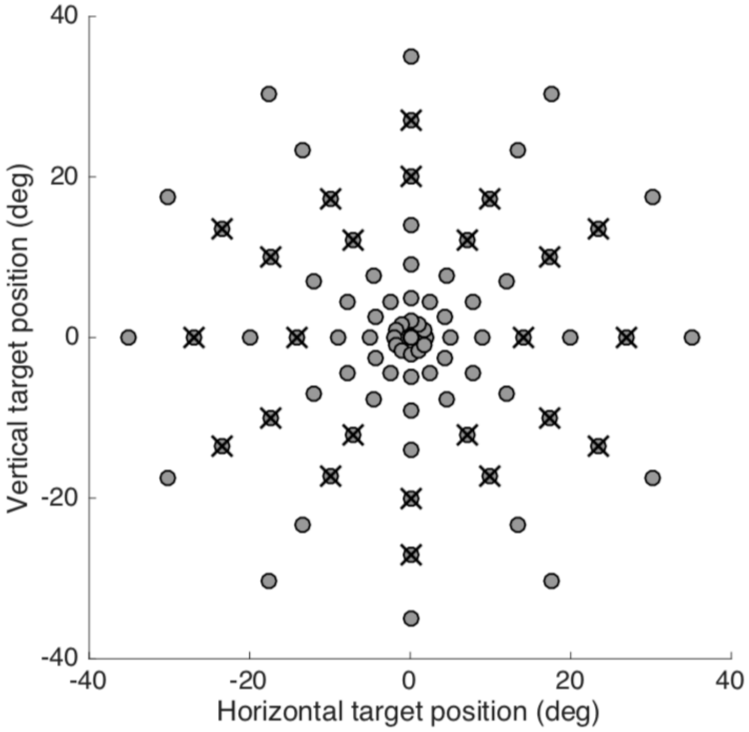

Figure S5. *Background visual display of the SNR experiments. The white-noise auditory background (at 60 dBA) is not shown (see Corneil et al., 2002). Grey dots: dimly-lit green LED locations (N=85); crosses: potential target locations, randomly selected in the experiment. Targets consisted of audio-visual (AV) stimuli (red LED and buzzer sound), V-only stimuli, or A-only stimuli. The target-sound intensities were selected from 39, 42, 48, and 54 dBA. All stimuli were randomly interleaved. We here report on the A-only targets (Figures 3, 4, and 6). The target locations for the LP experiment are depicted in Fig. 5A.*

S6. Alternative prior distributions

Clearly, when the shape of the prior distribution is left entirely free, any response pattern can in principle be fitted by the MAP decision rule. In the paper we opted for Gaussian distributions, not only for mere analytical convenience (Eqns. 5-9), but also because the response data seem to suggest normally distributed patterns (e.g. Fig. 1).

However, because in the experiments the stimuli were drawn from a finite target range, and especially in the SNR experiments this was made explicitly evident by means of the dim visual background (Fig. S5), one may wonder whether perhaps the assumed prior distribution may have reflected this imprinted target range. The two experiments (SNR and LP) employed target ranges: [-35, +35] deg and approximately [-90, +90] deg in azimuth and elevation.

In Fig. S6 we show the results of the gain-variability relationship of the MAP model for two situations: a uniform box prior in [-40, +40] deg, while targets were drawn from [-90, +90] deg (left), and from [-40,+40] deg (right). The MAP results for these box-priors are indicated by the orange symbols, and are compared to the MAP rule on a Gaussian prior, for which we here took a large standard deviation of 30 deg. It is immediately clear from these results that although the small box-prior on the right-hand side predicts a monotonic decrease of the gain with increasing response variance, qualitatively similar as seen in the data, the slope of this relationship is far too low, as the curve will intersect the G=0 axis at about +45 deg, rather than at 12 deg, as seen in the data. We conclude that the Gaussian prior, in combination with random posterior sampling, explains the data best.,

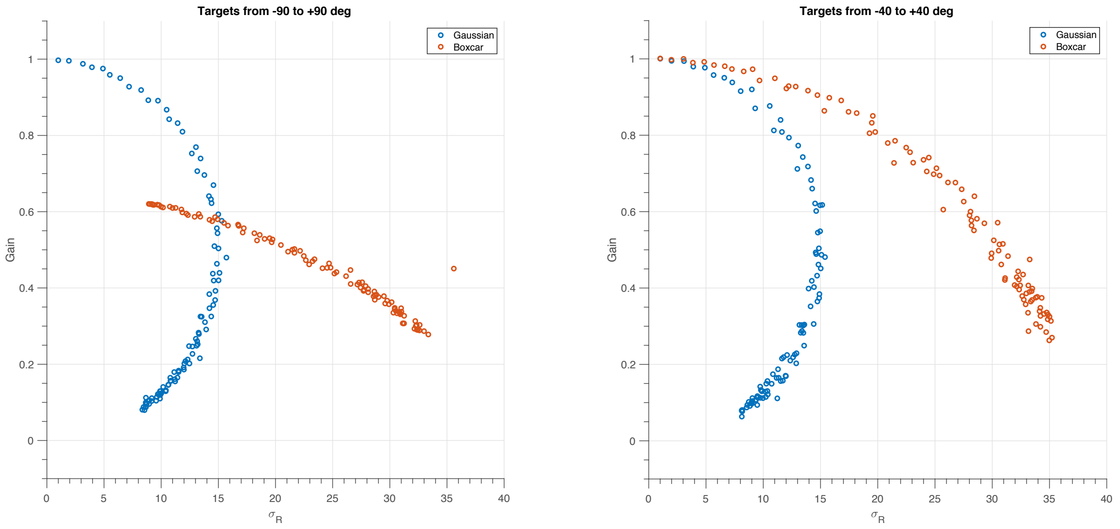

Figure S6: *Simulations of the accuracy-precision relationship for a MAP decision model when a box-car prior (orange symbols) of [-40,40] deg is chosen, in case of two different actual target ranges: [-90,+90] deg (left), or [-40, +40] deg (right). The box-car simulations are compared to a MAP decision rule with a Gaussian prior that has a standard deviation of 30 deg (blue*

symbols). Although the box-prior yield monotonically decreasing relations, they differ profoundly from the experimental data shown in Fig. 6.

S7. Regression results for the LP responses of all seven individual subjects

Figure 5 shows the pooled data from seven subjects (S6-S12). Here we provide the results from the individual subjects in the same format as Figure 5.

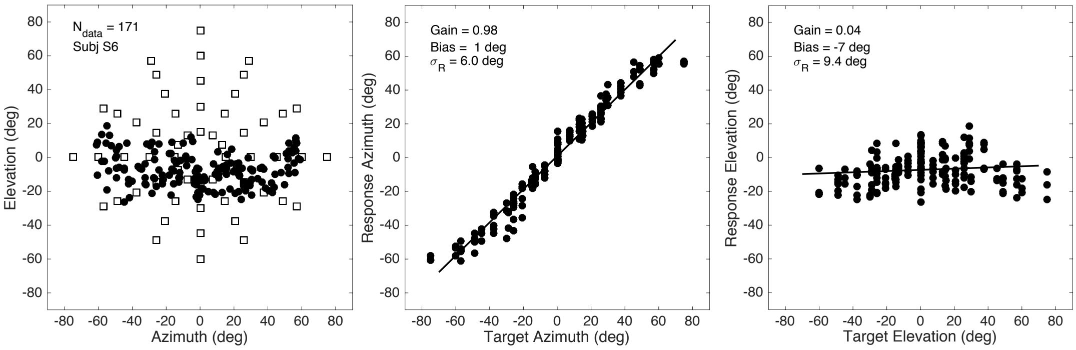

Figure S7-1: Response data from listener S6 to LP filtered noises (<1.5 kHz). Compare Fig. 5.

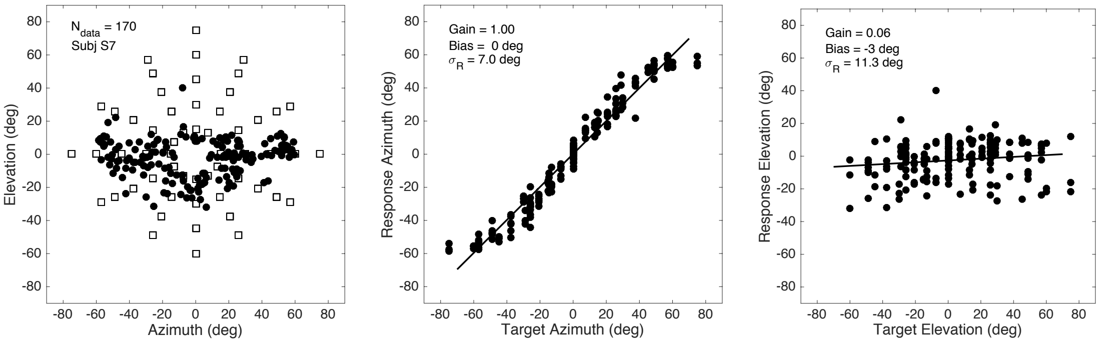

Figure S7-2: Response data from listener S7 to LP filtered noises (<1.5 kHz).

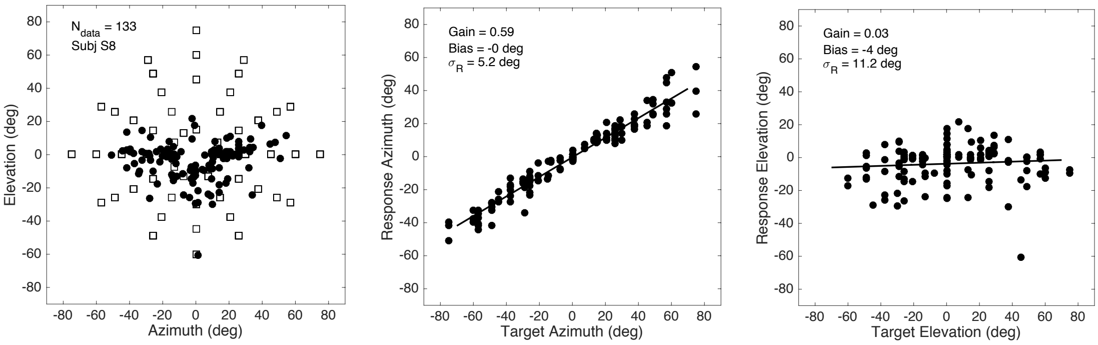

Figure S7-3: Response data from listener S8 to LP filtered noises (<1.5 kHz). Note the low gain for this listener, which falls more than 3σ from the mean (and therefore is not included in the analysis of Fig. 6A).

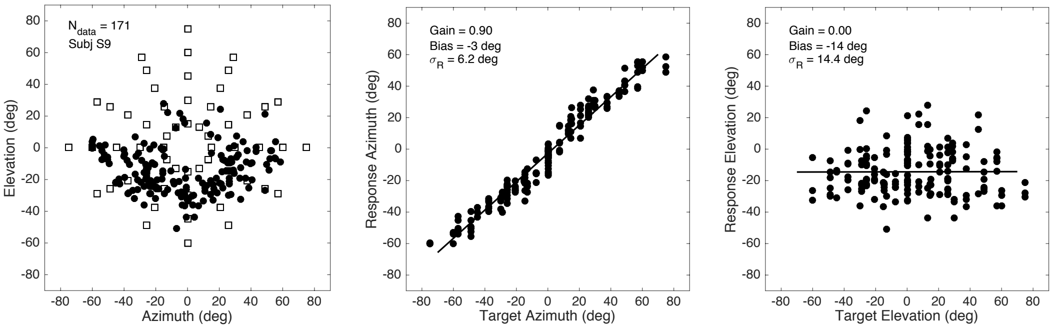

Figure S7-4: Response data from listener S9 to LP filtered noises (<1.5 kHz).

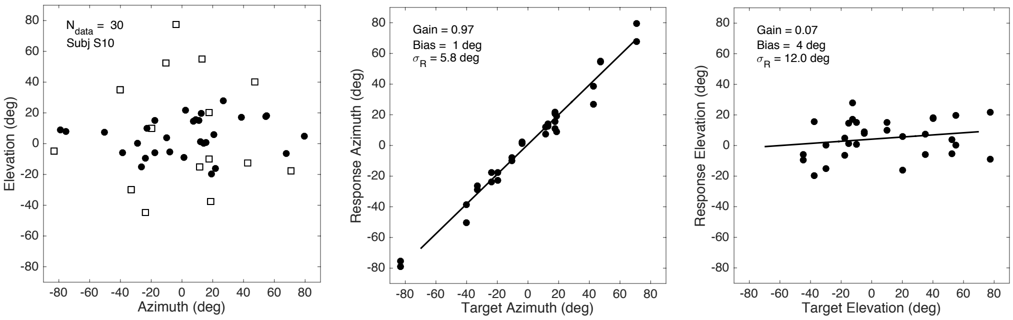

Figure S7-5: Response data from listener S10 to LP filtered noises (<3 kHz).

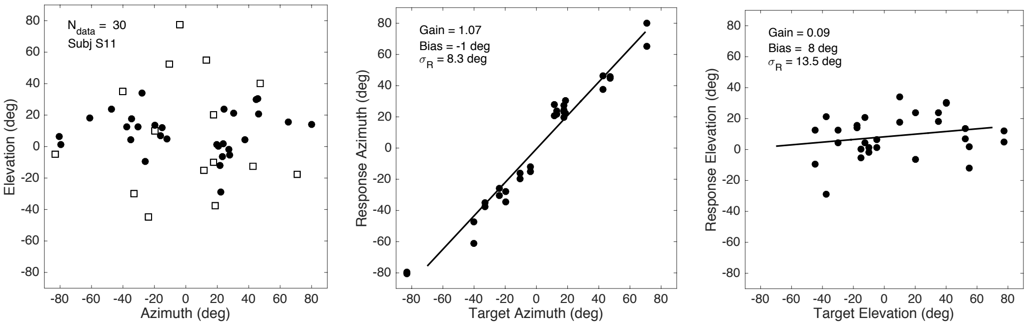

Figure S7-6: Response data from listener S11 to LP filtered noises (<3 kHz).

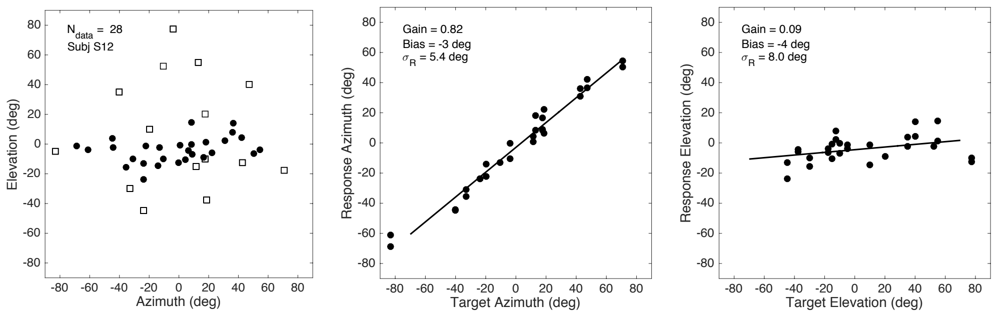

Figure S7-7: Response data from listener S12 to LP filtered noises (<3 kHz).
